# Bendamustine induces peripheral lymphodepletion in dogs with relapsed lymphoma

**DOI:** 10.1093/jvimsj/aalag283

**Published:** 2026-11-02

**Authors:** Jennifer A Lenz, Laurel Upton, Jonathan Ma, Mia M Talone, Brandon Peng, Lang Jiang, Audrey Ghanian, Dillon Didehvar, Ester Yang, Joseph A Fraietta, Matthew J Atherton

**Affiliations:** Department of Clinical Sciences and Advanced Medicine, School of Veterinary Medicine, University of Pennsylvania, Philadelphia, PA, United States; Department of Clinical Sciences and Advanced Medicine, School of Veterinary Medicine, University of Pennsylvania, Philadelphia, PA, United States; Department of Clinical Sciences and Advanced Medicine, School of Veterinary Medicine, University of Pennsylvania, Philadelphia, PA, United States; Department of Clinical Sciences and Advanced Medicine, School of Veterinary Medicine, University of Pennsylvania, Philadelphia, PA, United States; Department of Biomedical Sciences, School of Veterinary Medicine, University of Pennsylvania, Philadelphia, PA, United States; Department of Clinical Sciences and Advanced Medicine, School of Veterinary Medicine, University of Pennsylvania, Philadelphia, PA, United States; Department of Clinical Sciences and Advanced Medicine, School of Veterinary Medicine, University of Pennsylvania, Philadelphia, PA, United States; Department of Clinical Sciences and Advanced Medicine, School of Veterinary Medicine, University of Pennsylvania, Philadelphia, PA, United States; Department of Clinical Sciences and Advanced Medicine, School of Veterinary Medicine, University of Pennsylvania, Philadelphia, PA, United States; Department of Microbiology, Perelman School of Medicine, University of Pennsylvania, Philadelphia, PA, United States; Center for Cellular Immunotherapies, Perelman School of Medicine, University of Pennsylvania, Philadelphia, PA, United States; Department of Pathology and Laboratory Medicine, Perelman School of Medicine, University of Pennsylvania, Philadelphia, PA, United States; Department of Clinical Sciences and Advanced Medicine, School of Veterinary Medicine, University of Pennsylvania, Philadelphia, PA, United States; Department of Biomedical Sciences, School of Veterinary Medicine, University of Pennsylvania, Philadelphia, PA, United States

**Keywords:** bendamustine, canine lymphoma, lymphodepletion, preconditioning

## Abstract

**Background:**

Chimeric antigen receptor (CAR) T-cell therapy has improved outcomes for many human patients with B-cell lymphoma and provides a rational template for analogous canine patients. To date, the expansion, persistence, and activity of CAR T cells in dogs have been limited. Lymphodepletion supports CAR T-cell engraftment and antitumor activity. In human medical oncology, bendamustine has emerged as a well-tolerated lymphodepleting agent. However, prospective use of bendamustine for dogs with lymphoma has not been characterized.

**Hypothesis/Objectives:**

To determine the lymphodepleting activity and tolerability of bendamustine in dogs with relapsed lymphoma. Secondary objectives included monitoring serum cytokines and short-term changes in peripheral tumor burden.

**Animals:**

Eleven client-owned dogs with relapsed lymphoma.

**Methods:**

Luminescent assays were used to assess the direct cytotoxic activity of bendamustine against canine lymphoid cell lines*.* A prospective rapid dose-escalation pilot trial was undertaken in 11 dogs with relapsed lymphoma to evaluate tolerability while tracking peripheral lymphocyte counts, serum cytokine concentrations, and lymph node measurements.

**Results:**

Bendamustine induced concentration-dependent cytotoxicity in all cell lines tested. Two-dose bendamustine regimens up to 90 mg/m^2^ were generally well tolerated, with no dose-limiting toxicities observed, and resulted in peripheral lymphodepletion (pretreatment median, 880 cells/μL; posttreatment median, 310 cells/μL), an exploratory decrease in circulating interleukin-6 (pretreatment median, 5.32 pg/mL; posttreatment median, 1.33 pg/mL), and a short-term decrease in peripheral nodal burden in 8 of 11 dogs.

**Conclusions and clinical importance:**

Bendamustine was generally well tolerated and induced lymphodepletion in our cohort. These findings support further evaluation of bendamustine as a preconditioning agent before autologous canine CAR T-cell infusion.

## Introduction

The development of anti-CD19 chimeric antigen receptor T-cell (CAR T) therapy for aggressive B-cell lymphoma in people has emerged as a highly effective therapeutic approach.^1^^,^^2^ Because B-cell lymphoma is frequently encountered and often life-limiting in dogs, CAR T technology could be beneficial for veterinary patients.^3^^,^^4^ Prior work indicates that treatment of dogs with B-cell lymphoma using autologous anti-CD20 CAR T-cells is feasible, generally well tolerated, and recapitulates several clinical sequelae of CAR T therapy encountered in human medicine.^5^^,^^6^ However, hallmarks of CAR T activity, including CAR T expansion and persistence, B-cell aplasia, and sustained antitumor responses were not observed.

Studies of human CAR T patients indicate that inadequate engraftment of CAR T-cells represents a major cause of therapeutic failure, highlighting the importance of in vivo expansion and long-term persistence of infused cells in determining favorable treatment outcomes.^7–11^ Lymphodepletion using a combination of cyclophosphamide and fludarabine (Cy/Flu) for preconditioning before CAR T infusion has proven to be a crucial strategy for the successful expansion, persistence, and antitumor activity of infused CAR T-cells in human patients.^12–15^ Preconditioning chemotherapy before CAR T can deplete endogenous lymphocytes and circulating suppressive cells while modulating serum cytokines.^12–16^ Furthermore, improved responses to CAR T-cell therapy in patients with large B-cell lymphoma who had attained complete or partial responses to cytotoxic treatments immediately before CAR T infusion suggest that decreasing tumor burden may also contribute to CAR T-cell activity.^17^ Thus, preconditioning likely supports infused CAR T-cells through several host-level effects rather than through lymphocyte depletion alone.

Although utilization of Cy/Flu for CAR T preconditioning has been described in healthy dogs, the toxicity profile of these agents has limited routine use of this regimen in canine cancer patients.^18^ Bendamustine is a unique anticancer cytotoxic agent that is well tolerated in humans and, when used at a dosage of 90 mg/m^2^ given on consecutive days before CAR T infusion for treatment of B cell lymphomas, can provide a conditioning effect comparable to Cy/Flu, but with less associated toxicity.^19–21^ Because bendamustine has not been prospectively characterized in tumor-bearing dogs, our primary goals were to determine the lymphodepleting activity and tolerability of bendamustine in dogs with relapsed lymphoma. Secondary goals were to track changes in serum cytokines and short-term nodal burden in a prospective rapid dose-escalation pilot trial.

## Materials and methods

### Cytotoxicity

CLBL-1 (gift from Dr Barbara C. Rütgen), EO-1 (gift from Dr Sadatoshi Maeda), and GL-1 (gift from Dr Nicola Mason) cells were retrovirally transduced to express green fluorescent protein (GFP) and luciferase (Addgene 75021), and purified. Cells were grown in RPMI-1640 with GlutaMAX and 1 mM HEPES (Gibco), with 10% heat-inactivated fetal bovine serum and penicillin–streptomycin (Gibco), at 37°C, 95% relative humidity, and 5% CO_2_. Ten thousand cells were plated in triplicate in 96-well plates and cultured with bendamustine for 24 h. Wells used to define spontaneous and maximal cytotoxicity contained media and 0.5% Triton X-100 (Acros Organics), respectively. ONE-Glo luciferase reagent (Promega) was added to wells and luminescence was detected using a GloMax Discover reader (Promega). Cytotoxicity (%) per well was determined according to the equation:

100 × (spontaneous cytotoxicity relative light units [RLU] − bendamustine RLU)/(spontaneous cytotoxicity RLU − maximal cytotoxicity RLU).

Cells were routinely tested for mycoplasma and identity was confirmed using morphology and flow cytometry.

### Trial

Dogs with cytologically confirmed relapsed intermediate-to-large or large-cell lymphoma were eligible. Measurable peripheral lymphadenopathy, body weight ≥ 10 kg, and life expectancy ≥ 1 month were inclusion criteria. Severe or life-limiting comorbidities and concurrent corticosteroid use were exclusion criteria. The study began with 2 doses of bendamustine hydrochloride (Bendeka, Teva) given IV on consecutive days. Dogs were eligible to receive additional chemotherapy at completion or lymphoma progression. Blood was collected throughout the study for routine monitoring (Table 1). Cryopreserved (−80°C) serum samples were analyzed using the canine cytokine/chemokine premixed 13-plex kit (Millipore Sigma). Bendamustine-associated dose-limiting toxicities were defined as grade ≥ 3 non-hematologic events or grade ≥ 4 hematologic events according to Veterinary Cooperative Oncology Group (VCOG) guidelines.^22^ Peripheral disease burden was monitored using the sum of the longest submandibular, prescapular, and popliteal lymph node diameters. Day 8 change in peripheral burden (%) was defined as:

**Table 1:** Study timeline.

Visit number	1	2	3	4	5	6
***Day number***	*1*	*2*	*5*	*8*	*13-17*	*19-23*
**Physical exam and history**	X	X	X	X	X	X
**Lymph node measurement**	X	X	X	X	X	X
**Complete blood count**	X		X	X	X	
**Serum biochemistry**	X				X	
**Serum cytokine collection**	X		X	X		
**Bendamustine**	X	X				

(sum of nodal diameters at day 8 − baseline sum of nodal diameters)/baseline sum of nodal diameters × 100.

A −100% nodal change was assigned to dogs with normal nodal diameter and no detectable disease elsewhere. Approval was obtained from the University of Pennsylvania’s Institutional Animal Care and Use Committee (#807255), and the Privately Owned Animal Protocol committee (#632). Signed owner consent was obtained.

### Statistics

Half-maximal inhibitory concentration (IC50) was calculated by plotting % cytotoxicity against bendamustine concentration and performing nonlinear regression employing [Inhibitor] vs response—Variable slope (4 parameters) model. Two-tailed Wilcoxon matched-pairs signed-rank tests were used for paired comparisons. Analyses were conducted using GraphPad Prism v11. Significance was defined as *P* < .05 (* < .05; ** ≤ .01, and *** ≤ .001).

## Results

### Bendamustine is cytotoxic against canine lymphoid cell lines in vitro

To determine the cytotoxicity of bendamustine against canine lymphoid cancer cell lines in vitro we acquired a diffuse large B-cell lymphoma cell line (CLBL-1), an epitheliotropic T-cell lymphoma cell line (EO-1), and an acute leukemia cell line (GL-1). These cells were stably transduced with retrovirus to express luciferase and GFP before purification using fluorescence-activated cell sorting of GFP-labeled cells, thus enabling the performance of luminescent cytotoxicity assays (Figure S1). We documented cytotoxicity in all 3 modified cell lines after 24 h of exposure to a dilution gradient of bendamustine (Figure 1). The EO-1 cells were most sensitive to bendamustine (IC50 at 24 h of 11.82 μg/mL), followed by CLBL-1 (IC50 at 24 h of 17.56 μg/mL), with GL-1 cells being least sensitive to killing by bendamustine (IC50 could not be accurately estimated at 24 h). Taken together, these data demonstrate direct in vitro susceptibility of multiple malignant canine lymphoid cell lines to bendamustine.

**Figure 1:**
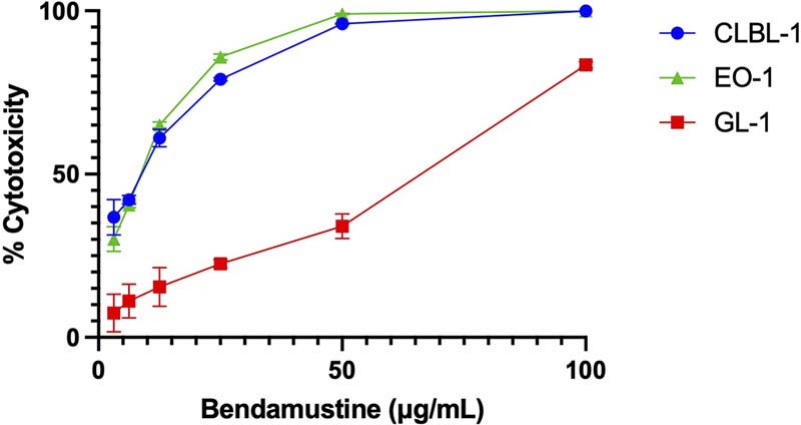
Cytotoxicity curves of 3 canine lymphoid cell lines after exposure to a dilution series of bendamustine for 24 h. Representative plot of 1 of 2 independent experiments is shown.

### Population of relapsed canine lymphoma patients

Although bendamustine has not been prospectively characterized in canine cancer patients, our in vitro cytotoxicity data alongside safety and activity data for this drug in human patients provided a rationale to evaluate bendamustine for use in canine lymphoma patients. As such, we designed a prospective, rapid dose-escalation, pilot study to determine the tolerability and lymphodepleting activity of bendamustine in relapsed canine lymphoma patients. Secondary goals included documenting short-term lymph node changes and tracking serum cytokines. Eleven dogs were recruited, and all dogs had cytologically confirmed intermediate-large cell (5 dogs) or large cell (6 dogs) lymphoma. The median age of the dogs was 6 years (range, 3-11) and included 7 neutered males, 3 spayed females, and 1 intact male. There were 7 mixed-breed dogs and 1 each of Border Collie, Boxer, Mastiff, and Great Dane. Median weight at the first dose of bendamustine was 29.2 kg (range, 19.8-82.7). All dogs had cell lineage determined and 8 dogs had B-cell disease, and 3 dogs had T-cell disease. All patients had relapsed after cyclophosphamide, doxorubicin, vincristine, prednisone (CHOP)-based chemotherapy regimens. Additional rescue regimens prescribed to a subset of dogs before bendamustine included rabacfosadine, lomustine, procarbazine, prednisone (LPP); lomustine, vincristine, procarbazine, prednisone (LOPP); lomustine, vinblastine, procarbazine, prednisone (LVPP); and L-asparaginase (Table S1). This cohort enabled a preliminary assessment of tolerability, peripheral lymphodepletion, cytokine changes, and short-term lymph node changes after bendamustine in dogs with lymphoma.

### A single cycle of bendamustine is generally well tolerated in dogs with relapsed lymphoma

Because bendamustine use in tumor-bearing dogs is not well characterized, we designed a rapid dose-escalation study comprising 2 IV doses of bendamustine administered approximately 24 h apart followed by sample collection and toxicity and disease burden monitoring for an intended period of 3 weeks (±2 days; Table 1). Complete blood counts and serum biochemistry profiles were obtained at baseline before the first dose of bendamustine. In all dogs, CBCs were repeated at days 5 and 8 of the study and approximately one week thereafter for those dogs that remained on the trial. Serum biochemistry profiles were repeated for patients that remained on the trial for 5 visits or more at visit 5 (corresponding to days 13-17). The initial starting dosage of bendamustine was 45 mg/m^2^ given twice, 1 day apart, and was escalated in 7.5 mg/m^2^ increments until a dosage of 82.5 mg/m^2^ was reached. Intrapatient dose escalation was not performed. At 82.5 mg/m^2^, cohorts were expanded to a traditional 3 + 3 design, as this dosage was approaching the lymphodepleting dosage of 90 mg/m^2^ described for human patients with relapsed or refractory large B-cell lymphoma undergoing CAR T therapy.^20^ No dose-limiting toxicities were observed, and all dogs received the complete cycle of bendamustine at the intended dosage. Adverse events deemed possibly, probably, or definitely attributable to bendamustine monotherapy were primarily related to hematologic and gastrointestinal disturbances (Table 2). Two patients developed signs presumably associated with nausea during the first of the 2 infusions and these signs resolved with supportive care. Grade 3 changes to appetite, defined as alterations in appetite lasting for longer than 2 weeks, occurred in 3 dogs, but because alterations to appetite were reported before enrollment in all 3 patients and anorexia was not documented, these events were deemed unrelated to bendamustine (Table 2). In summary, 2-dose bendamustine regimens up to the CAR T lymphodepletion dosage used in humans generally were well tolerated in our pilot cohort. The maximum tolerated dose was not established because the study was designed to evaluate feasibility and lymphodepletion rather than escalation beyond 90 mg/m^2^.

**Table 2:** VCOG toxicity grading by total number of events for bendamustine.

	Grade				
Adverse event	1	2	3	4	5
***Blood/bone marrow***					
**Low hemoglobin**	5^B^; 4^D^				
**Low PCV**	6^B^; 2^D^				
**Neutropenia**	2^E^	3^E^	2^E^		
**Thrombocytopenia**	2^B^; 1^E^	1^D,*^			
***Cardiac general***					
**Sinus tachycardia**	2^B^				
***Constitutional clinical signs***					
**Lethargy/fatigue**	1^B^; 2^D^	2^B^			
**Fever**	1^B^				
**Weight loss**	2^D^				
***Dermatologic/skin***					
**Alopecia**	1^A^				
**Erythema**	2^A^				
**Hyperpigmentation**	2^A^				
***Gastrointestinal***					
**Appetite, altered**	1^B^; 1^D^	1^B^	3^A^		
**Diarrhea**	1^E^	3^E^			
**Nausea/ptyalism**		2^E^			
**Vomiting**	2^E^				
***Metabolic/laboratory***					
**ALT, high**		1^B^			
**Calcium, low**	1^A^				
**Creatinine, high**	1^C^				
**Glucose, high**	1^A^				
**Potassium, low**	1^B^; 1^D^				
**Phosphorous, low**	1^A^; 1^C^				
**Sodium, low**	1^D^				
***Neurology***					
**Spinal cord compression**		1^A^			

Adverse event attribution: A. Unrelated: The adverse event is clearly not related to bendamustine. B. Unlikely: The adverse event is doubtfully related to bendamustine. C. Possible: The adverse event may be related to bendamustine. D. Probable: The adverse event is likely related to bendamustine. E. Definite: The adverse event is clearly related to bendamustine.

^*^Platelet clumping noted on slide evaluation.

Abbreviations: ALT = alanine aminotransferase; VCOG = Veterinary Cooperative Oncology Group.

### Bendamustine induces peripheral lymphodepletion, is associated with lower circulating interleukin-6, and produces short-term reductions in nodal burden

To determine the peripheral immunomodulatory properties of bendamustine given in the absence of corticosteroids, we compared lymphocyte counts at baseline to the lowest count measured on day 5 or day 8 of the study. Across the cohort, a significant decrease in circulating lymphocyte count was documented from a pretreatment median count of 880 cells/μL to a nadir of 310 cells/μL (Figure 2). Serum cytokines were analyzed on day 5 and day 8 of the study in all patients except patient one, for which the day 8 sample was omitted, and thus we compared circulating cytokine concentrations between baseline and day 5 only. Of the 13 cytokines analyzed, we observed an unadjusted decrease in serum interleukin-6 (IL-6) concentrations from a pretreatment median of 5.32 to 1.33 pg/mL at day 5, with no significant changes detected in the remaining cytokines (Figure 3; Figure S2). Finally, to determine the impact of bendamustine treatment on tumor burden, we measured peripheral lymph node diameters at baseline, and determined the percentage change in summed peripheral lymph node diameter at day 8. Tumor burden increased in 3 dogs and decreased in 8 dogs, with 2 of these dogs reaching normal measured peripheral lymph node diameter which represented a −100% day 8 nodal change (Figure 4). In summary, bendamustine induced peripheral lymphodepletion, was associated with lower serum IL-6 concentrations, and produced short-term decreases in nodal burden in relapsed canine lymphoma patients.

**Figure 2:**
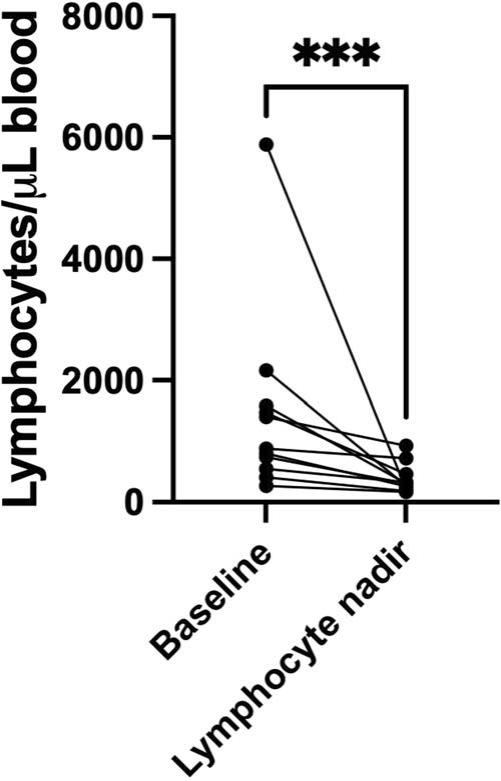
Comparison of circulating lymphocyte counts in relapsed canine lymphoma patients before bendamustine and at the lowest observed count on day 5 or day 8. *P* = .001. Abbreviation: OR = overall response.

**Figure 3:**
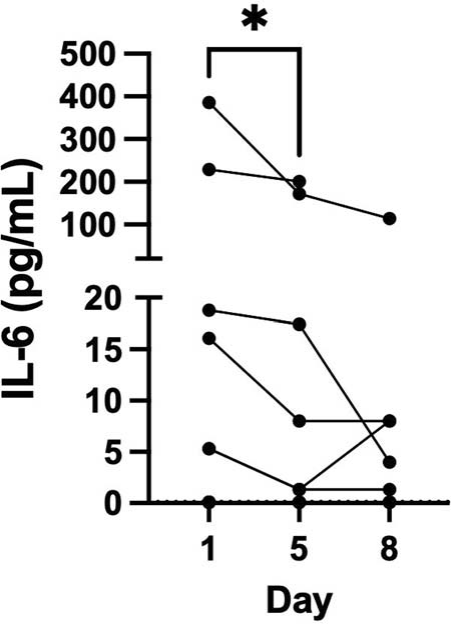
Exploratory comparison of circulating IL-6 concentrations in relapsed canine lymphoma patients before and after bendamustine treatment. Statistical analyses were performed between prebendamustine and day 5. *P* = .031. Abbreviation: IL = interleukin.

**Figure 4:**
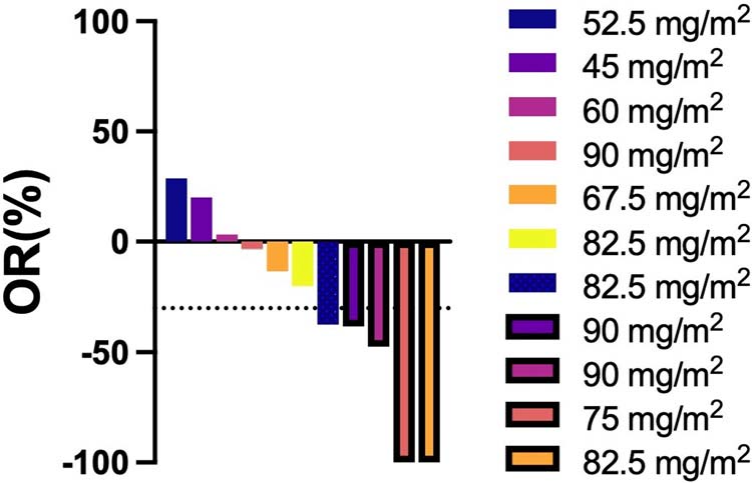
Percent change in summed peripheral nodal disease burden at day 8 compared with baseline measurements. Negative values indicate reduction in nodal burden; dashed line at −30%; −100% indicates normalization of measured peripheral nodes with no detectable disease elsewhere.

## Discussion

Bendamustine generally was well tolerated in our cohort of dogs with relapsed lymphoma at dosages up to those used for CAR T lymphodepletion in humans. Beyond tolerability, bendamustine induced peripheral lymphodepletion, was associated with a decrease in serum IL-6 concentrations in exploratory cytokine analyses, and produced short-term decreases in peripheral nodal burden in most dogs. Collectively, these data support further evaluation of bendamustine as a candidate lymphodepleting agent for CAR T studies in dogs rather than as definitive evidence of clinical efficacy.

Beyond describing the application of bendamustine as a lymphodepleting candidate, our data provide preliminary evidence to support further investigation of bendamustine as a cytotoxic drug for dogs with lymphoma. Our in vitro cytotoxicity data identified potent killing of both a diffuse large B-cell lymphoma cell line and an epitheliotropic cell line, and this result was directionally consistent with the short-term decreases in lymph node size observed in patients. The IC50s for the 2 most sensitive canine lymphoid cell lines are consistent with those reported in human lymphoid cell lines, but these in vitro exposures should not be interpreted as pharmacokinetic targets in dogs.^23^^,^^24^ Although the lymphoma cell lines were relatively sensitive to killing by bendamustine, the GL-1 acute leukemia cell line was more resistant. This finding is consistent with prior data comparing IC50s of GL-1 and CLBL-1 cells when exposed to other drugs, including alkylators such as cyclophosphamide and lomustine.^25^

The in vitro sensitivity of the EO-1 epitheliotropic lymphoma line is notable given that the alkylator lomustine is frequently prescribed for this indication, yet reported complete response rates after lomustine-containing regimens for dogs with epitheliotropic lymphoma are approximately 30%.^26^^,^^27^ In addition to standard alkylating activity, bendamustine has additional functions that differentiate it from other alkylators such as having purine analog-like properties, thus providing a hypothetical advantage over other drugs in the same class.^19^^,^^23^^,^^28^ Additional studies to define the maximal tolerated dosage and ability to combine this agent with other antineoplastic agents, including corticosteroids, for both relapsed disease and challenging cases of epitheliotropic disease, will be needed before determining the full potential of bendamustine as a traditional cytotoxic agent in dogs with lymphoma.

Lymphodepletion, described as the induction of lymphopenia before CAR T infusion, is now an established part of the clinical practice of treating human B-cell lymphoma patients with approved autologous CAR T treatments.^29^ Despite the clear importance of lymphodepletion for the activity of CAR T cells in humans, limited data are available in canine patients. The magnitude of peripheral lymphodepletion in our canine patients (from pretreatment median of 880 cells/μL to a posttreatment median of 310 cells/μL) is broadly aligned with that documented previously in people prescribed 90 mg/m^2^ of bendamustine (from pretreatment median of 600 cells/μL to a posttreatment median of 190 cells/μL).^20^ Notably, this magnitude of peripheral lymphodepletion was achieved in a cohort of dogs that had previous exposure to multi-agent chemotherapy without observing dose-limiting toxicities.

Additional observations relevant to future CAR T studies were detected, but they should be considered exploratory. In most dogs, we documented a decrease in measured peripheral lymph node burden one week after bendamustine. This timing is relevant because it overlaps with the anticipated window for CAR T infusion and early in vivo expansion in future studies in dogs. In people with large B-cell lymphoma, lower tumor burden or effective cytoreduction before CAR T infusion has been associated with improved outcomes.^17^ Potential explanations include the reduction of an immunosuppressive hostile tumor microenvironment, which could foreseeably act as an obstacle to CAR T activity. Alternatively, this association with improved outcomes may also simply represent a subset of disease that is less refractory to multiple therapeutic approaches.^14^^,^^17^^,^^29^ Additional mechanistic studies are required to determine whether this effect is causative or not.

Finally, we also noted a decrease in serum IL-6 concentrations, which is biologically relevant but should be interpreted cautiously because the cytokine panel was exploratory. Interleukin-6 is considered a key mediator of the potentially life-threatening cytokine release syndrome in humans after CAR T cell therapy.^30^ Increased serum IL-6 concentration has also been reported in a canine patient after autologous CAR T therapy.^6^ Bendamustine preconditioning has been associated with a lower incidence of cytokine release syndrome in humans undergoing CAR T therapy, further supporting interest in this regimen.^20^ Increased IL-6 concentrations have also been associated with worse outcomes in both human and canine lymphoma patients.^31–33^ Taken together, lower serum IL-6 concentration after bendamustine could be clinically advantageous in the context of CAR T therapy.

Our study had some limitations. It was an uncontrolled 11-dog pilot study with heterogeneous lymphoma subtypes, prior treatments, and dosages; follow-up was limited to a single cycle. Lymphodepletion was assessed using peripheral absolute lymphocyte counts rather than immune subsets, and cytokine analyses were exploratory.

In conclusion, treatment with bendamustine was feasible and generally well tolerated in our cohort and induced peripheral lymphodepletion at dosages relevant to planned CAR T preconditioning in dogs. Additional studies should define the maximum tolerated or recommended biologic dosage, pharmacokinetics, immune subset effects, combination strategies, and safety immediately before autologous CAR T-cell infusion.

## Supplementary Material

Supplementary_Materials_aalag283

## Off-label antimicrobial declaration

The authors declare no off-label use of antimicrobials.

## Institutional animal care and use committee or other approval declaration

Approval was obtained from the University of Pennsylvania’s Institutional Animal Care and Use Committee (#807255), and the Privately Owned Animal Protocol committee (#632). Signed owner consent was obtained for all dogs enrolled in the trial.

## Abbreviations

CAR: chimeric antigen receptor
CHOP: cyclophosphamide, doxorubicin, vincristine, prednisone
Cy/Flu: cyclophosphamide and fludarabine
GFP: green fluorescent protein
IC50: half-maximal inhibitory concentration
IL-6: interleukin-6
LOPP: lomustine, vincristine, procarbazine, prednisone
LPP: lomustine, procarbazine, prednisone
LVPP: lomustine, vinblastine, procarbazine, prednisone
OR: overall response
VCOG: Veterinary Cooperative Oncology Group
