# Supplementary material for: Bendamustine induces peripheral lymphodepletion in dogs with relapsed lymphoma
Source: J Vet Intern Med. 2026 Nov 2;40(6):aalag283. doi: 10.1093/jvimsj/aalag283 (PMC13725755; doi:10.1093/jvimsj/aalag283)
Supplement: Supplementary file 1 [file supplementary_materials_aalag283.zip › Supplementary_Figures_caption_aalag283.docx]

**Figure S1** Flow cytometry plots of three wild type canine lymphoid cell lines and the same lines after γ-retroviral engineering to stably express a cassette encoding GFP and luciferase after purification by flow sorting of GFP expressing cells. Gates reveal % frequency of GFP expressing cells gated on live singlets.

**Figure S2** Comparison of circulating cytokine concentrations in relapsed canine lymphoma patients before and after bendamustine treatment. Statistical analyses were performed on the complete dataset between pre-bendamustine and day five. ns= not significant.
