# Supplementary material for: Bendamustine induces peripheral lymphodepletion in dogs with relapsed lymphoma
Source: J Vet Intern Med. 2026 Nov 2;40(6):aalag283. doi: 10.1093/jvimsj/aalag283 (PMC13725755; doi:10.1093/jvimsj/aalag283)
Supplement: Supplementary file 1 [file supplementary_materials_aalag283.zip › Table S1.docx]

**Table S1. Individual patient data.** MC (male castrated); FS (female spayed); FC (flow cytometry); PARR (PCR for antigen receptor rearrangement)

| **Patient** | **Bendamustine dose (mg/m^2^**) | **Sex** | **Breed** | **Age (yrs)** | **Weight (kgs)** | **Cytological diagnosis** | **Cell lineage** | **Lineage test** | **Prior treatment** |
| --- | --- | --- | --- | --- | --- | --- | --- | --- | --- |
| 1 | 45 | MC | Mixed | 10 | 23.5 | Large cell | B cell | FC & PARR | CHOP |
| 2 | 52.5 | MC | Mixed | 6 | 29.2 | Intermediate-large | B cell | FC | L-CHOP rabacfosadine |
| 3 | 60 | MC | Mixed | 6 | 27.6 | Large cell | B cell | FC | CHOP × 2 rabacfosadine |
| 4 | 67.5 | MC | Boxer | 5 | 39.2 | Intermediate-large | T cell | FC | CHOP  LPP |
| 5 | 75 | FS | Mixed | 5 | 28.8 | Large cell | B cell | FC | CHOP |
| 6 | 82.5 | MC | Mixed | 5 | 29.2 | Large cell | B cell | PARR | CHOP rabacfosadine LOPP |
| 7 | 82.5 | M | Mastiff | 5 | 82.7 | Intermediate-large | B cell | FC | CHOP |
| 8 | 82.5 | MC | Mixed | 11 | 29.7 | Intermediate-large | T cell | FC | L-CHOP |
| 9 | 90 | FS | Mixed | 7 | 39.2 | Intermediate-large | T cell | FC | CHOP |
| 10 | 90 | FS | Border Collie | 6 | 19.8 | Large cell | B cell | FC | CHOP |
| 11 | 90 | MC | Great Dane | 3 | 61.8 | Large cell | B cell | FC | L-spar  CHOP  LVPP |
